# Trends and disparities in place of death among patients with cholangiocarcinoma: A two-decade analysis

**DOI:** 10.1017/S147895152510031X

**Published:** 2025-06-20

**Authors:** Imran Qureshi, Charmi Patel, Steven Rella, Evan Botterman, Yazan Abboud, Ritik Goyal, Kaveh Hajifathalian, Paul Gaglio, Ahmed Al-Khazraji

**Affiliations:** 1 Department of Internal Medicine, Rutgers New Jersey Medical School, Newark, NJ, USA; 2 School of Medicine, Rutgers New Jersey Medical School, Newark, NJ, USA; 3 Division of Gastroenterology and Hepatology, Rutgers New Jersey Medical School, Newark, NJ, USA

**Keywords:** Cholangiocarcinoma, healthcare disparities, health policy and outcomes, cancer epidemiology, advanced care planning

## Abstract

**Objectives:**

Cholangiocarcinoma (CCA) is the second most lethal primary hepatic malignancy. It has been well-reported that most cancer patients prefer to die at home or in a hospice facility. However, there is limited data on the place of death for CCA patients. We evaluated trends and disparities in place of death for patients with CCA from 1999 to 2020.

**Methods:**

Using the CDC WONDER database (1999–2020), we calculated the frequency of CCA deaths at home/hospice and the average annual percentage change (AAPC) over this period stratified by race, age, gender, and region. We employed logistic regression to assess for associations between these variables and place of death for patients whose death was attributed to CCA.

**Results:**

Among 140,422 deaths, a rise in deaths occurred in home/hospice facilities compared to inpatient medical or nursing facilities across all variables examined. Blacks and individuals ≥ 85 had the highest proportion of deaths outside of home/hospice. However, Blacks showed the highest AAPC (8.56%) in home/hospice deaths, followed by Asians (AAPC 8.44%). In contrast, individuals aged 45–54 saw the lowest AAPC (4.27%). Non-whites were less likely to die at home/hospice, with Blacks demonstrating the lowest adjusted odds ratio (aOR 0.64). Those ≥ 85 were less likely to die in home/hospice (aOR 0.78), whereas individuals aged between 55–64 (aOR 1.11) and 65–74 (aOR 1.12) had increased odds of dying in these settings. Patients from the Western region were the most likely to die at home/hospice (aOR 1.04).

**Significance of results:**

Our study highlights disparities in place of death of patients with CCA amongst races, regions, and ages. Non-whites, extremes of ages, and patients from the Northeast have disproportionately poor outcomes in terms of end-of-life care in the US. These findings emphasize the need for efforts to address sociodemographic disparities in end-of-life care to improve patient-centered health outcomes.

## Introduction

End-of-life care prioritizes a person’s comfort and quality of life in the final months, weeks, and days of their life. The practice of palliative and hospice care has improved the management of terminally ill persons nearing the end of life. The primary purpose of end-of-life care is to manage a person’s pain and psychological and spiritual needs and provide support for their family and friends. This can be optimally provided at a specialized facility or home. Where palliative care occurs depends on the intensity of the care required, but patient preference is also a deciding factor. Most people prefer to die at home or in a nursing facility. It has been reported in the medical literature that the quality of life of those receiving palliative care is higher in those with good social support, including family members and friends, and when a person does not have to change their environment, such as receiving hospice at home (Gomes et al. 2013; Higginson et al. 2002; Tang et al. 2004). However, home hospice care is not always an option for some people, and many factors play a role in whether a patient can receive this care, including severity of illness, socioeconomic status, brevity of the terminal phase of disease, and hesitancy of family. Hospice and palliative care enhance the quality of life in individuals nearing the end of their life; however, the application of this care model can vary significantly across different disease and patient populations.

Cholangiocarcinoma (CCA) is a malignant tumor arising from the biliary system and is considered the second fatal primary liver cancer after hepatocellular cancer. CCA is rare,accounting for 3% of gastrointestinal tumors, and has an overall incidence of less than 2/100,000 (Kirstein and Vogel 2016). CCA carries a high mortality rate, with as low as ten percent for a 5-year survival rate (Everhart and Ruhl 2009). CCA represents a significant burden of disease, accounting for around 20% of deaths from hepatobiliary cancers, which cause approximately 13% of the total cancer mortality worldwide (Everhart and Ruhl 2009; Kirstein and Vogel 2016). Surgery is the only definitive treatment method, but most individuals with CCA are not good surgical candidates, primarily secondary to late-stage diagnosis, and it is estimated that less than 20% of people qualify for surgical resection (Bath and Pawlik 2023; Patel 2011; Zamani and Fatima 2023). In addition, although liver transplantation is a potential option to manage patients with CCA, most patients with CCA do not qualify for this procedure. As CCAs are associated with high mortality rates and limited treatment options, the use of palliative and hospice care plays a key role in disease management and end-of-life care.

New research has shown that people prefer to die at home or in a hospice facility when compared to a hospital setting (Gomes et al. 2013; Tang et al. 2004). Palliative and comfort care is dependent on the patient’s disease state and demographics, so it is worthwhile to understand aspects related to the provision of end-of-life care in the context of their disease, age group, socioeconomic status, race, gender, and ethnicity (Chun et al. 2022; Robison et al. 2023). There is limited research on the disparities in the location of death home or hospice versus hospital among patients with terminal CCA, as well as on evolving trends and practices in end-of-life care for this population. This study aims to assess the trends and disparities in the place of death among patients with a diagnosis of CCA.

## Methods

### Data source

Our cross-sectional study was conducted using data provided by the Centers for Disease Control and Prevention (CDC) WONDER (Wide-ranging Online Data for Epidemiologic Research) dataset. The National Center for Health Statistics manages this dataset and contains deidentified data pertaining to mortality and population, including demographics and the underlying cause of death as noted on the death certificates of the entire US population, including the fifty states and the District of Columbia. The data is publicly available and accessible through WONDER’s online platform. Data provided includes place of residence, age, race, ethnicity, gender, year, cause-of-death, injury intent and injury mechanism, drug/alcohol-induced causes, urbanization categories, place of death, month and weekday of death, and whether an autopsy was performed. In order to maintain confidentiality, values representing fewer than ten persons are suppressed when this dataset is queried (Centers for Disease Control and Prevention 2024).

### Cohort selection

We queried the WONDER dataset from 1999 to 2020 using the International Statistical Classification of Diseases and Related Health Problems, Tenth Revision (ICD-10) codes C22.1, C24.0, C24.1, C24.8, and C24.9 to identify those whose underlying cause of death was CCA. These patients were stratified by place of death, which included inpatient, outpatient/ER, dead on arrival, status unknown, nursing home/long-term care, other, decedent’s home, or hospice facility. Of note is that hospice facilities were reported as places of death only after 2003. We further classified these patients by race – American Indian (or Alaska Native), Asian (or Pacific Islander), Black (or African American), or White, gender – male or female, age groups – < 45, 45–54, 55–64, 65–74, 75–84 or ≥ 85 years, and census regions – Northeast, Midwest, South or West.

### Statistical analysis

For our study, we divided the place of death into two broad categories: death at home or hospice facility and death elsewhere (inpatient, outpatient/ER, dead on arrival, status unknown, nursing home/long-term care, or other). Early-onset CCA was defined as CCA being the cause of death in patients aged less than 45 years of age. We divided patient characteristics by home/hospice versus death elsewhere and summarized these findings in a table depicting counts and percentages. We also looked at the trends of percentages of deaths in home/hospice versus elsewhere for patients with CCA as the cause of death between 1999 and 2020. Furthermore, we looked at the trend of the percentage of deaths at home/hospice by race, gender, age groups, and census regions and visualized these findings in the form of line graphs. To quantify and compare these findings, we calculated the average annual percentage change (AAPC) of death at home/hospice from 1999 to 2020 and demonstrated these findings as a forest plot. To do so, we ran a Poisson regression while adjusting for the fact that the total number of deaths varies from year to year. Additionally, we utilized chi-squared tests to analyze categorical data for significance. To assess predictors of the place of death for patients with CCA, we employed frequency-weighted univariate logistic regression followed by multivariate logistic regression using the variables of race, gender, age groups, and census regions. We reported our findings in the form of odds ratios (OR) and adjusted odd ratios (aOR), along with their confidence intervals and p-values, for univariate logistic regression and multivariate logistic regression, respectively, and summarized our findings in the form of a forest plot. For logistic regression, we used our variables’ most observed values as references: whites for race, males for gender, 75–84 years for age groups, and south for census region. We considered a p-value of less than 0.05 as statistically significant. All statistical analysis was done using StataNow/MP 18.5 for Mac (StataCorp LLC, Texas, USA).

## Results

### Patient characteristics

The study cohort consisted of 140,422 individuals (Table 1) with a roughly even distribution of both sexes: 70,225 individuals were male, and 70,197 were females. In total, 120,915 participants were White, 12,241 were Black, 6,691 were Asian, and 575 were American Indian. Of all the races, only Black individuals had more deaths in other settings compared to home or at a hospice facility, with 5,712 (46.7%) in home or hospice settings and 6,529 (53.3%) in other settings. When stratified into age groups, there were 3,123 among individuals aged less than 45, 10,628 deaths amongst those between ages 45 and 54, 27,117 amongst those aged 55 to 64 years, 39,128 deaths between 65 and 74 years of age, 39,435 in patients aged 75 to 84 years of age, and 20,991 in those aged over 85.

**Table 1.** Frequency and percentages of patient demographic characteristics divided by home-hospice versus death elsewhere between 1999 and 2020 of patients with CCA as cause of death

	Home-Hospice Deaths (n = 78262)	Deaths Elsewhere (n = 62160)	Total Deaths (n = 140422)
**Race**			
White	68670 (56.8%)	52245 (43.2%)	120915
Black	5712 (46.7%)	6529 (53.3%)	12241
Asian	3565 (53.3%)	3126 (46.7%)	6691
American Indian	315 (54.8%)	260 (45.2%)	575
**Gender**			
Male	39346 (56.0%)	30879 (44.0%)	70225
Female	38916 (55.4%)	31281 (44.6%)	70197
**Age Groups**			
<45 years	1727 (55.3%)	1396 (44.7%)	3,123
45−54 years	5891 (55.4%)	4737 (44.6%)	10628
55−64 years	15617 (57.6%)	11500 (42.4%)	27117
65−74 years	22744 (58.1%)	16384 (41.9%)	39128
75−84 years	21901 (55.5%)	17534 (44.5%)	39435
>85 years	10382 (49.5%)	10609 (50.5%)	20991
**Region**			
South	26891 (57.7%)	19736 (42.3%)	46627
West	18444 (59.0%)	12843 (41.0%)	31287
Midwest	18126 (54.7%)	14995 (45.3%)	33121
Northeast	14801 (50.4%)	14586 (49.6%)	29387

The Northeast region, with a total of 29,387 deaths, had the highest proportion of deaths in other settings: 14,801 (50.4%) in home or hospice and 14,586 (49.6%) in other settings. 33,121 deaths were reported in the Midwest, amongst which 18,126 (54.7%) were at home or hospice and 14,995 (45.3%) in other settings. The highest number of deaths was reported in the Southern region, with a total of 46,627 deaths, 26,891 (57.7%) home/hospice deaths, and 19,736 (42.3%) deaths in other settings. There were 31,287 recorded deaths in the Western region, of which 18,444 (59.0%) were at home or hospice, and 12,843 (41.0%) were in other settings.

### AAPC

**Figure 1.**
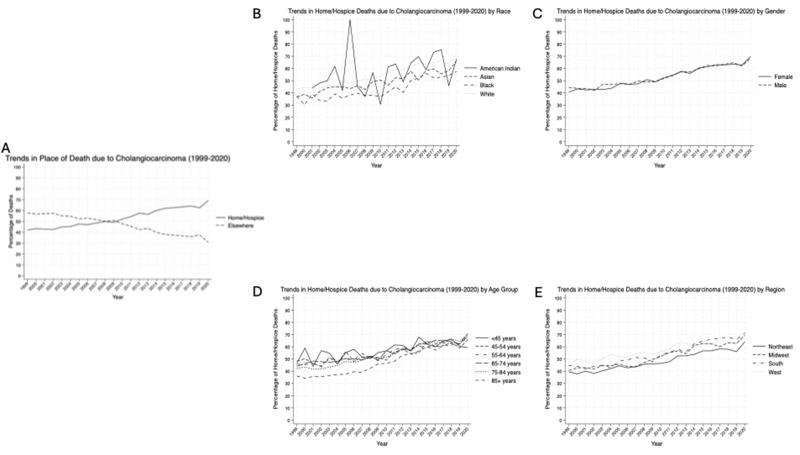
Time trends in death at home or hospice between 1999 and 2020 for all CCA patients (A) and patient-specific demographics including race (B), gender (C), age group (D), and region (E).

**Figure 2.**
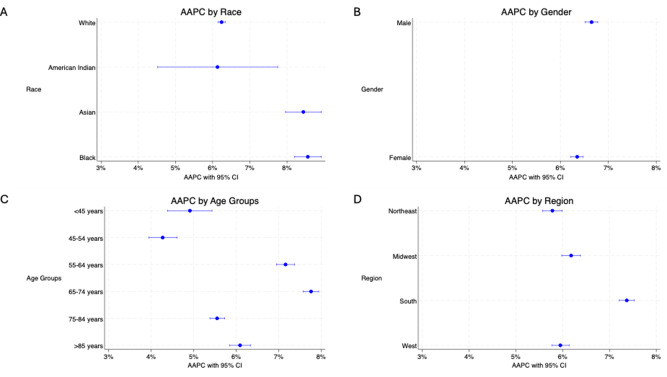
AAPC of death at home or hospice facility between 1999 and 2020 in patients with CCA of different races (A), genders (B), age groups (C), and regions (D).

Our trend graphs showed an overall trend toward increased home/hospice deaths (Figure 1A), though this data varied when stratified by race, gender, age groups, and census regions (Table 2). We calculated the overall AAPC of 6.55% (95% CI 6.46–6.64, p < 0.001), demonstrating an overall increased trend toward home and hospice deaths. For race, we saw a statistically significant increase in AAPC (Figures 1B and 2A) for home and hospice deaths for each of the groups with White individuals increasing by 6.24% (95% CI 6.15–6.34, p < 0.001), American Indian individuals increasing by 6.13% (95% CI 4.52–7.75, p < 0.001), Asian individuals by 8.44% (95% CI 7.96–8.92, p < 0.001), and Black individuals having the highest AAPC at 8.56% (95% CI 8.20–8.92, p < 0.001). The AAPC for both genders (Figures 1C and 2B) were also positive, and similar increases were seen between the groups, with males increasing by 6.65% (95% CI 6.52–6.78, p < 0.001) and females increasing by 6.35% (95% CI 6.22–6.47, p < 0.001). Each of the age groups also saw AAPC increase (Figures 1D and 2C), with less than 45 year olds increasing at a rate of 4.91% (95% CI 4.38–5.43, p < 0.001), 45–54 years old increasing by 4.27% (95% CI 3.94–4.61, p < 0.001), 55–64 years old increasing by 7.16% (95% CI 6.95–7.37, p < 0.001), 65–74 years old increasing 7.76% (95% CI 7.58–7.94, p < 0.001), 75–84 years old increasing 5.55% (95% CI 5.38–5.73, p < 0.001), and 85 + years old increasing 6.09% (95% CI 5.84–6.34, p < 0.001). Each of the regions also saw an AAPC increase (Figures 1E and 2D), with the Northeast increasing by 5.78% (95% CI 5.57–5.99, p < 0.001), the Midwest by 6.18% (95% CI 5.98–6.38, p < 0.001), the South by 7.37% (95% CI 7.21–7.53, p < 0.001), and the West by increasing 5.95% (95% CI 5.77–6.14, p < 0.001).

**Table 2.** Time-trends, in the form of average annual percentage change (AAPC) with 95% confidence intervals (CI) and p-values of the deaths at home-hospice between 1999 and 2020 of patients with CCA as cause of death along with univariate and multivariable logistic regression reported with odds ratio (OR) and adjusted odds ratio (aor), respectively, along with their 95% confidence interval (CI) and p-values for predictors of home-hospice versus death elsewhere

	AAPC	95% CI	P-value	OR	95% CI	P-value	aOR	95% CI	P-value
**Overall**	6.55%*	(6.46−6.64)	<0.001						
**Race**									
White	6.24%*	(6.15−6.34)	<0.001	1	(Reference)	(Reference)	1	(Reference)	(Reference)
Black	8.56%*	(8.20−8.92)	<0.001	0.67*	(0.64−0.69)	<0.001	0.64*	(0.62−0.66)	<0.001
Asian	8.44%*	(7.96−8.92)	<0.001	0.87*	(0.83−0.91)	<0.001	0.81*	(0.77−0.85)	<0.001
American Indian	6.13%*	(4.52−7.75)	<0.001	0.92	(0.78−1.09)	0.332	0.79*	(0.67−0.93)	0.004
**Gender**									
Male	6.65%*	(6.52−6.78)	<0.001	1	(Reference)	(Reference)	1	(Reference)	(Reference)
Female	6.35%*	(6.22−6.47)	<0.001	0.98*	(0.96−1.00)	0.026	1.01	(0.98−1.03)	0.626
**Age Groups**									
<45 years	4.91%*	(4.38−5.43)	<0.001	0.99	(0.92−1.07)	0.797	0.99	(0.92−1.06)	0.694
45−54 years	4.27%*	(3.94−4.61)	<0.001	1	(0.95−1.04)	0.843	1.01	(0.97−1.06)	0.525
55−64 years	7.16%*	(6.95−7.37)	<0.001	1.09*	(1.05−1.12)	<0.001	1.11*	(1.07−1.14)	<0.001
65−74 years	7.76%*	(7.58−7.94)	<0.001	1.11*	(1.08−1.14)	<0.001	1.12*	(1.09−1.15)	<0.001
75−84 years	5.55%*	(5.38−5.73)	<0.001	1	(Reference)	(Reference)	1	(Reference)	(Reference)
85+ years	6.09%*	(5.84−6.34)	<0.001	0.78*	(0.76−0.81)	<0.001	0.78*	(0.75−0.81)	<0.001
**Region**									
South	7.37%*	(7.21−7.53)	<0.001	1	(Reference)	(Reference)	1	(Reference)	(Reference)
West	5.95%*	(5.77−6.14)	<0.001	1.05*	(1.02−1.09)	<0.001	1.04*	(1.01−1.07)	0.016
Midwest	6.18%*	(5.98−6.38)	<0.001	0.89*	(0.86−0.91)	<0.001	0.86*	(0.84−0.89)	<0.001
Northeast	5.78%*	(5.57−5.99)	<0.001	0.74*	(0.72−0.77)	<0.001	0.74*	(0.71−0.76)	<0.001

* Indicates statistically significant findings.

### Logistic regression analysis

**Figure 3.**
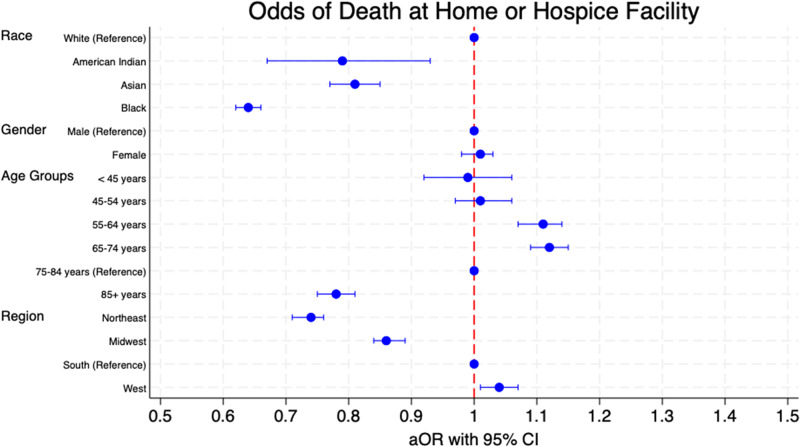
Adjusted odds ratios (aor) with 95% confidence intervals of death at home or hospice facility patients of different races, genders, age groups, and regions.

We found Asian individuals (OR 0.87, 95% CI: 0.83–0.91, p < 0.001) and Black individuals (OR 0.67, 95% CI: 0.64–0.69, p < 0.001) were less likely to die at home or in a facility when compared to White individuals. This was the case even when adjusting for covariates for Asian individuals (aOR 0.81, 95% CI: 0.77–0.85, p < 0.001) and Black individuals (aOR 0.64, 95% CI: 0.62–0.66, p < 0.001). For American Indian individuals, the unadjusted analysis did not show a significantly lower likelihood of dying at home or in hospice facilities (OR 0.92, 95% CI 0.78–1.09, p = 0.332); however, after controlling for other variables, the adjusted odds ratio for American Indians significantly decreased to 0.79 (95% CI 0.67–0.93, p = 0.004) indicating a reduced likelihood of home and hospice care deaths (Table 2, Figure 3).

We noted a significant reduction in the likelihood of death in home or hospice care for females (OR 0.98, 95% CI 0.96–1.00, p = 0.026). However, after adjusting the data, it was shown that there was no statistically significant difference in the odds of death when comparing different settings (aOR 1.01, 95% CI 0.98–1.03, p = 0.626).

Our analysis also demonstrated that those older than 85 were significantly less likely to die in home or hospice care (OR 0.78, 95% CI 0.76–0.81, p < 0.001). The data continued to show a statistically significant decrease for this age group when performing multivariate logistic regression (aOR 0.78, 95% CI 0.75–0.81, p < 0.001). For age groups 55–64 years and 65–74 years, there was a statistically significant increased odds of death at home/hospice for both univariate (OR 1.09, 95% CI 1.05–1.12, p < 0.001) and (OR 1.11, 95% CI 1.08–1.14, p < 0.001), respectively) and multivariate logistic regression (aOR 1.11, 95% CI 1.07–1.14, p < 0.001) and (aOR 1.12, 95% CI 1.09–1.15, p < 0.001), respectively). The age groups that did not show a statistically significant difference in the place of death compared to the reference group in either the unadjusted or adjusted data were individuals less than 45 years old, with unadjusted results (OR 0.99, 95% CI 0.92–1.07, p = 0.797), and adjusted results (aOR 0.99, 95% CI 0.92–1.06, p = 0.694), and the 45–54 years old age group, with unadjusted results (OR 1.00, 95% CI 0.95–1.04, p = 0.843), and adjusted results (aOR 1.01, 95% CI 0.97–1.06, p = 0.525).

There was a statistically significant decrease in the likelihood of home-hospice death in the Northeast region (OR 0.74, 95% CI 0.72–0.77, p < 0.001) and the Midwest region (OR 0.89, 95% CI 0.86–0.91, p < 0.001). These findings were statistically significant even when adjusting for covariates for both the Northeast (aOR 0.74, 95% CI 0.71–0.76, p < 0.001) and the Midwest (aOR 0.86, 95% CI 0.84–0.89, p < 0.001). However, compared to the Southern, those from the Western were more likely to be at home or hospice (OR 1.05, 95% CI 1.02–1.09, p < 0.001). This statistical significance was preserved when adjusting for covariates (aOR 1.04, 95% CI 1.01–1.07, p < 0.001).

## Discussion

The results of this study demonstrate an overall increased trend toward death at home or in hospice care between 1999–2020, with an average annual percent change (AAPC) of 6.55%. However, subgroup analysis reveals significant disparities in the location of death when comparing different population groups, regions, and age groups with CCA. Notably, there are decreased odds of dying at home among American Indian, Black, and Asian subgroups when compared to the White individuals, with Blacks demonstrating the lowest likelihood of dying at home. Blacks were the only racial group that had a lower overall number of deaths at home or in a hospice facility than other facilities. At the same time, Blacks showed the highest AAPC of deaths at home or hospice facilities between 1999 and 2020. In terms of geographical variations for CCA deaths in the U.S., the Southern region and those in the Northeast and Midwest regions exhibited the lowest likelihood of dying at home or hospice facilities. In contrast, those in the Western region demonstrated the highest odds of dying at home or hospice facilities. The Northeastern region showed equivalent rates of deaths either at home or in a hospice facility. Interestingly, individuals aged 85 years or older were the least likely to die at home or hospice. Of all the age groups, only this group had a higher number of deaths outside home or hospice facility. The lowest AAPC was seen amongst those aged 45–54, followed by those under 45. These findings emphasize the need for more personalized care to address the cultural, regional, and age-related factors that influence end-of-life care preferences and practices, particularly among racial minorities, extremities of age, and those pertaining to certain geographical areas.

Our findings both corroborate existing literature and reveal novel insights into end-of-life care, highlighting important trends and disparities when assessing sociodemographic and geographic attributes. The growing number of patients with CCA opting for hospice care at the time of death, compared to inpatient settings or nursing facilities, is a pattern consistent with a broader trend. A similar trend has been reported in England, where home and hospice deaths from cancer have risen since 2005 (Gao et al. 2013). Likewise, Korea has experienced a notable increase in home deaths for cancer patients following the introduction of insurance coverage for home-based hospice care (Yun et al. 2023). This shift toward hospice and home deaths, along with a decline in inpatient hospital deaths, may reflect several factors. First, the ubiquitous emphasis on advance care planning has allowed patients to express their end-of-life preferences earlier, resulting in care that better aligns with their wishes – often favoring home or hospice deaths surrounded by loved ones (Khan et al. 2014). Improvements in palliative care practices and the decreasing stigma around death in the United States may also contribute to this trend.

In patients with CCA, we observed that American Indian, Asian, and Black individuals were less likely to die at home or in a hospice facility compared to their white counterparts. Although limited research specifically addresses how racial disparities affect the place of death in CCA, a study by Chino et al. found that Black, American Indian, and nonwhite Hispanic cancer patients were significantly less likely to utilize hospice care than white patients (Chino et al. 2018). Together, these findings suggest potential barriers to home or hospice deaths among minority groups. This may stem from reduced family support, financial or environmental challenges in accessing hospice care, or implicit bias affecting provider referrals. Furthermore, Black patients often present with CCA at a more advanced stage than white patients (Munir et al. 2023). This leads to accelerated health deterioration and higher rates of hospital deaths due to limited time and worsening prognosis. While our study reflects a general increase in hospice and home deaths, significant racial disparities persist.

There were notable regional differences in hospice utilization across the U.S. We observed that patients with CCA in the Northeast and Midwest were less likely to die at home or in a hospice facility compared to those in the South. Despite the Northeast’s leadership in legislated palliative care access – such as New York’s Palliative Care Information Act, which mandates that patients be informed about palliative care options – this did not correlate with higher hospice utilization in the region. In contrast, other areas, particularly the South, may lag in legal mandates and available services. However, given that CCA is an aggressive cancer with a poor prognosis, the Northeast’s higher density of advanced medical centers may contribute to these findings, explaining why there were increased rates of in-hospital deaths. Our results could reflect a higher prevalence of advanced treatments in these regions, inadvertently leading to more hospital deaths. For example, a study by Uhlig et al. in the *Annals of Surgical Oncology* found that the Middle Atlantic region, which includes states in the Northeast, had the highest surgery rates for intrahepatic CCA, indicating a more aggressive surgical approach (Uhlig et al. 2019). This could lead to increased complications and risk of subsequent hospital deaths. Similarly, our findings align with a study done by Connor et al., which also demonstrated higher hospice use in the South compared to the Northeast and Midwest. In another study by Virnig et al., rates of hospice use were over 10-fold higher in Florida than in Maine. In comparison, the West has even greater rates of hospice use compared to the South. The Western regions are known to have fewer hospital beds per capita (Hansen et al. 2002). The higher percentage of deaths occurring at home in the West correlates with a lower availability of in-hospital beds. Physician perspectives also play a crucial role in shaping patient awareness and acceptance of hospice care. Data on geographic differences in physician attitudes toward hospice are limited, but further research that explores how these perspectives vary across regions and influence hospice utilization in patients with CCA would be valuable.

The results of the current study show an important trend in the place of death among different age groups. We noticed a lower AAPC of hospice use or home death amongst younger patients compared to their older counterparts. Prior research indicates that younger patients are more likely to die in the hospital secondary to more aggressive treatments that often require hospital-based care (Falchook et al. 2017). This is likely because younger patients may be better surgical candidates and have fewer comorbid conditions. In contrast, we noted a significant increase in the rates of home or hospice death amongst those aged 55–74. Research by Cagle et al. found that older age is a significant predictor of hospice utilization among cancer patients (Cagle et al. 2020). Additionally, the literature suggests that older patients with primary liver cancer, including CCA, have a shorter time to hospice enrollment (Fukui et al. 2018). However, our findings show lower hospice utilization among patients over 85 years of age compared to those in late middle age. This discrepancy may reflect higher rates of postoperative complications in elderly patients undergoing major hepatectomy or interventional radiology procedures for CCA (de la Fuente Sg Bk and Scarborough 2013; Maeda et al. 2022), which can lead to prolonged hospital stays and increased mortality. These patients were also likely sicker, more debilitated, and had fewer resources for hospice. Further investigation into subcategorizing age groups based on the aggressiveness of treatment received could provide additional insights.

The odds of hospice or home death compared to hospital death among males versus females with CCA were similar. The observed lack of difference in gender and place of death is consistent with other studies that examine hospice use across various cancer diagnoses (Virnig et al. 2002). Although a study by Mojtahedi et al. suggested that females with extrahepatic CCA may be more likely to receive palliative care services compared to males, specific rates of hospice utilization by gender were not provided in this study (Mojtahedi et al. 2021).

We identified some limitations with our study, mostly pertaining to the dataset. Firstly, the WONDER dataset relies on death reporting by healthcare professionals, and some cases may have been missed. Secondly, the underlying cause of death reported in the WONDER dataset reflects the cause of death listed on the death certificate, which may not always be accurate and is open to interpretation. This may lead to an under-identification of patients who had CCA as the cause of death. Of note, Meino et al. reported that patients with cancer as an underlying cause of death tend to have an accurate death certificate (Mieno et al. 2016); these observations may not be accurate when applied to a larger database. Additionally, we expect the differences in cause of death reporting to remain constant over the years, allowing us to compare the groups adequately. The location of death reported may not always be accurate, such as when patients are being palliatively treated at home or in a hospice facility and are transferred to a medical facility right before their death. Furthermore, some patients may be treated palliatively in a medical facility, especially if prolonged survival is not anticipated. Moreover, hospice facilities as places of death were not introduced into the WONDER dataset until 2003. The CDC also suppresses values that involve less than ten individuals. To circumvent this, for trend graphs and AAPC, we requested data only divided by place of death and each of the variables, race, gender, age groups, and census regions. We did not employ this technique for logistic regression in order to maintain the same data across univariate and multivariate analyses. Finally, the WONDER dataset lacks the granularity to report additional patient or disease factors such as socioeconomic status, access to care, insurance status, malignancy stage, or treatment received. Further studies are needed to assess the effect of the aforementioned factors on place of death for patients with CCA.

The strengths of our study are several; our analysis not only highlights the disparities in the location of death for patients with CCA, but it also demonstrates trends in palliative care utilization and variation across different sociodemographic factors. The inequality that exists between non-White and White populations in place of death from CCA is strongly evidenced in our study. We hope that these results will positively impact the discussions around and management of end-of-life care in racial minorities with CCA. Education about the goals and benefits of hospice care with non-White populations will allow enhanced decision-making that aligns with the patient’s goals of care. More research is warranted to discover and address the barriers to hospice care that exist among non-White populations in the United States. Additional findings demonstrating the disparities between regions and age groups show a significant disproportion in end-of-life care in the United States, regardless of race. The healthcare costs associated with these disparities are striking. Many studies have shown that the cost of dying in the hospital is dramatically more costly than dying at home. Hoverman et al. observed that when analyzing patients with cancer, dying in hospital is twice as costly as dying at home, with an average cost in the final month of life of $20,113 vs. $10,803, respectively (Hoverman et al. 2020). The emotional and physical costs of dying in the hospital can be alleviated by improving access to home hospice care. Wright et al. demonstrated that cancer patients who die in the hospital setting experience more physical and emotional distress than those who die at home (Wright et al. 2010). Dying at home impacts multiple aspects of a patient’s final moments of life. Our study has demonstrated a clear discrepancy in place of dying among specific races, regions, and age groups in patients with CCA and has important implications for public health stakeholders in addressing barriers to and potential improvement in the utilization of home hospice care for those with CCA.
